# A Western-Style Breakfast Induces a More Pro-Inflammatory Postprandial Response and Promotes Greater Macrophage Lipid Accumulation Compared to a Mediterranean-Style Breakfast in Obese and Normal-Weight Individuals

**DOI:** 10.3390/nu18040672

**Published:** 2026-02-18

**Authors:** Alejandro Matamoros-Domínguez, Laura Sinausia, Gisela Pérez-Muñoz, Juan Manuel Espinosa-Cabello, Aída García-González, Ana Rodríguez-Rodríguez, José María Castellano, Elena María Yubero-Serrano, Emilio Montero, Javier S. Perona

**Affiliations:** 1 Department of Food and Health, Campus Universida Pablo de Olavide, Building 46, Instituto de la Grasa-CSIC, Spanish National Research Council, 41013 Seville, Spain; alejandro.matamoros@ig.csic.es (A.M.-D.); lsnieva@gmail.com (L.S.); ipchanged29@gmail.com (G.P.-M.); juanmespinosa@ig.csic.es (J.M.E.-C.); aida.garcia@csic.es (A.G.-G.); jmcas@ig.csic.es (J.M.C.); elena.yubero@ig.csic.es (E.M.Y.-S.); 2 University Hospital Virgen del Rocío, Andalusian Regional Health Service, 41013 Seville, Spain; swroder_an@hotmail.com (A.R.-R.); emiliomonteroromero@gmail.com (E.M.); 3 CIBER Physiopathology of Obesity and Nutrition (CIBEROBN), Institute of Health Carlos III, 28029 Madrid, Spain

**Keywords:** postprandial metabolism, triglyceride-rich lipoproteins, dietary fat, inflammation, obesity

## Abstract

Background and objectives: Since postprandial lipid metabolism has emerged as a risk factor for cardiovascular disease, the quality of dietary fat may have a crucial role in atherogenesis and metabolic inflammation. In this study, we propose that the quality of dietary fats and the metabolic status of individuals modulate postprandial triglyceride-rich lipoprotein (TRL) composition and the response of macrophages to TRL. Methods: Randomized controlled crossover trial in the postprandial phase in 12 normal-weight adults and 12 adults with obesity. Each participant consumed both a Western-style (WB) and a Mediterranean-style (MB) breakfast in separate sessions, containing butter or olive oil as the fat source, respectively. Blood samples were collected at baseline (0 h), 2 h, and 4 h postprandially, and TRL were isolated and used to treat THP-1 macrophages. Results: The intake of the WB led to higher concentrations of inflammatory-related markers, particularly in individuals with obesity, and resulted in a higher content of saturated fatty acids and lower of monounsaturated fatty acids in TRL compared to the MB. Staining TRL-treated macrophages with Oil Red O revealed substantial lipid accumulation, which was more pronounced in cells cultured with 4 h TRL from individuals with obesity. This was also evidenced by upregulation of gene expression of lipoprotein uptake receptors following the consumption of the WB. Conclusions: Consumption of a WB led to a more pro-inflammatory postprandial profile and promoted greater lipid accumulation in macrophages, particularly in individuals with obesity, compared to a MB. These findings highlight the importance of fat quality in meals for cardiovascular risk management, especially in populations with obesity.

## 1. Introduction

According to the World Health Organization (WHO), obesity is a chronic complex disease defined by excessive fat deposits, which increases the risk of type 2 diabetes, metabolic syndrome, and coronary heart disease, and is becoming a global health crisis [1]. Moreover, obesity has been linked with several intestinal disturbances, specifically related to the mechanisms of lipid absorption and elevation of postprandial triglyceridemia. Current evidence supports that postprandial hypertriglyceridemia is a significant risk factor for cardiovascular disease and is strongly associated with visceral adiposity. Since a modern lifestyle involves frequent meals, resulting in prolonged postprandial lipemia, this persistent increase in triglycerides (TGs) is associated with adverse metabolic outcomes [2].

Triglyceride-rich lipoproteins (TRLs), comprising chylomicrons (CMs), very low-density lipoproteins (VLDLs) and their remnant particles, are the primary carriers of circulating TG. Their atherogenicity is explained by the conversion of nascent particles to their remnants, transcytosis into the arterial intima, and subsequent deposition of lipids in arterial walls, leading to foam cell formation and release of pro-inflammatory intermediaries (cytokines, interleukins, and adhesion molecules) [3]. Most evidence regarding the atherogenicity of TRL is focused on the total amount of TG in the particles and not on their fatty acid (FA) composition. Since current dietary recommendations include a reduction in saturated FA (SFA) and an emphasis on increasing mono- (MUFA) and polyunsaturated (PUFA) FA, a better understanding of the mechanisms of action of dietary FA on human lipoproteins is a priority [4]. Previous studies support the idea that the quality and quantity of a meal’s fat are key factors in determining the magnitude of postprandial response in terms of inflammation [5,6]. However, further research is required to elucidate the effects of different lipid sources on inflammatory markers, receptor expression, and TRL FA composition during the postprandial phase in individuals with obesity.

The widespread adoption of Western dietary habits and the globalization of food markets have significantly transformed eating behaviors worldwide, contributing to a rise in non-communicable diseases. In contrast, other dietary patterns have been associated with health benefits. The Mediterranean diet is characterized by several cardioprotective foods, among which olive oil stands out as a central element due to its high content of MUFA, which helps reduce cardiovascular risk factors [7,8,9]. These benefits have been evidenced in many long-term randomized controlled trials [10,11]. In contrast, unlike fermented and low-fat dairy products, butter, due to its SFA content, has been associated with increased LDL cholesterol concentration and a higher risk of cardiovascular disease [12,13].

In light of current evidence, this study aims to investigate the postprandial composition of TRL in obese and normal-weight subjects following the consumption of either a Western-style (WB) or Mediterranean-style (MB) breakfast, rich in SFA and MUFA, respectively. Specifically, we seek to characterize the FA composition of TRLs, evaluate their impact on intracellular lipid accumulation and composition in macrophages, and assess changes in receptor gene expression and circulating inflammatory adipokines. We hypothesize that both dietary fat quality and the metabolic status of individuals determine TRL composition and functionality, thereby influencing postprandial inflammatory and metabolic response.

## 2. Materials and Methods

### 2.1. Study Design and Participants

The study was designed as a randomized, controlled, and crossover postprandial trial (ClinicalTrials.gov NCT01518803 on 23 January 2012). The protocol and statistical analysis plan can be accessed at ClinicalTrials.gov. Participants were healthy adult men (20–40 years old) with no gastrointestinal or metabolic disorders, no dietary supplement or pharmacological treatment, and serum lipid profiles within reference ranges, recruited in Seville (Spain) between February and March 2011, through advertisements at universities and social networks. The trial was reported in accordance with the CONSORT, and the completed checklist is provided as Supplementary Material.

**Figure 1:**
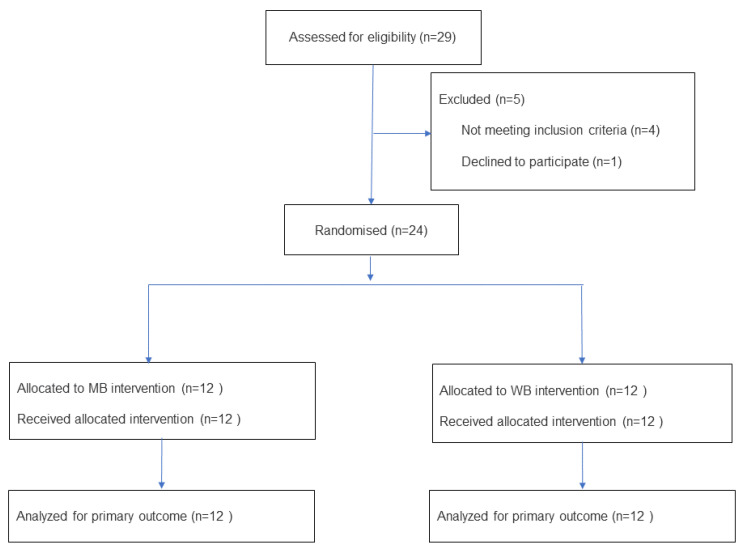
Flowchart of participants according to the CONSORT statement.

The primary quantitative variable of interest was the serum TG concentration. We postulated that the difference in TG between arms (MB and WB) would be at least 20%. We tested this null hypothesis of unilateral contrast, comparing two paired means (repeated in a group), with a confidence level of 90% (α = 0.01) and a power of 90% (β = 0.01), assuming participant losses of less than 10% to be tolerable. We used the “GRANMO Sample Size Calculator” software version 8.0 determining a sample size of 12 volunteers (Figure 1).

Subjects being treated with drugs for any kind of disease, suffering from chronic diseases, not willing to participate in the study or having participated in another clinical study in the preceding 3 months were excluded. Participants were classified into two groups based on their body mass index (BMI): normal weight (BMI 20–25 kg/m^2^) and obese (BMI > 30 kg/m^2^). Participants were instructed to refrain from consuming alcohol and tobacco during the day before the start of the experiment.

### 2.2. Ethical Considerations

All procedures were conducted following the principles of the Declaration of Helsinki (1975, revised in 2000), the Council of Europe Convention on Human Rights and Biomedicine, and the UNESCO Universal Declaration of Bioethics and Human Rights. A study protocol involving human participants was prepared and approved by the Ethical Committee at San Cecilio Hospital (registry 201160000048258, 25 January 2011). All volunteers provided written informed consent after receiving both verbal and written information about the study. Participation was voluntary, and subjects were informed of their right to withdraw at any time.

### 2.3. Dietary Intervention and Settings

After a 12 h overnight fast, participants underwent anthropometric and blood pressure measurements. Baseline blood samples were obtained, and immediately, participants were randomly assigned by the principal investigator (JSP) to one of the two experimental breakfasts by simple randomization using sequential numbers. The MB included olive oil (55 g), crushed fresh tomato (20 g), commercial sliced white bread (3 slices per participant; 23.7 g per slice), skimmed milk (200 mL), and orange juice (250 mL). The olive oil used in the MB was provided by Oleícola El Tejar S.C.A. (Córdoba, Spain), and its FA composition was supplied by the manufacturer and is detailed in Table 1. The WB consisted of commercial butter (55 g), 3 slices of white bread, and skimmed milk (200 mL) with cocoa powder (20 g), which were administered by the research team. The FA composition of the butter was analyzed at the Instituto de la Grasa-CSIC, and it is shown in Table 1. The nutritional composition of both experimental meals is summarized in Supplementary Table S1, based on the nutritional information provided on the packaging. During the postprandial period, blood samples were collected at 2 and 4 h. These time points were selected based on previous studies, as they correspond to the expected peak serum TG concentration and the lowest one, respectively [14]. In a second session, conducted after a washout period of one week, baseline blood samples were collected, and the participants who had initially consumed the MB received the WB, and vice versa. No harms or unintended events were observed.

### 2.4. Blood Biochemistry

Blood was obtained via the cubital vein using a Smartsite™ system equipped with a Vacutainer^®^ (Vacutainer^®^, Meylan Cedex, France). Blood samples were processed and analyzed in the Biochemistry and Clinical Analysis Laboratory of the Virgen del Rocío University Hospital to determine serum glucose and insulin, as well as lipid profiles, following standard procedures. Additionally, blood aliquots were sent to Instituto de la Grasa-CSIC, where serum was obtained by centrifugation at 3000 rpm for 8 min at 15 °C using a swinging-bucket rotor centrifuge (Eppendorf Centrifuge 5804R, Hamburg, Germany) and stored at −80 °C in a continuously monitored biobank until the isolation of TRL. The work with TRL was conducted prospectively between 2021 and 2023. This timeline enabled the integration of original clinical outcomes with subsequent mechanistic investigations. All samples from both study arms were analyzed concurrently in the same batch for each assay to ensure internal comparability.

### 2.5. Adipokine, MCP-1 and Ceruloplasmin Serum Concentrations

Serum concentrations of leptin, adiponectin, and ceruloplasmin were measured by ELISA using kits from AssayPro (St. Charles, MO, USA), following the manufacturer’s instructions. MCP-1 levels were determined with an ELISA kit from Boster (Pleasanton, CA, USA), also according to the manufacturer’s protocol.

### 2.6. Characterization of Postprandial TRL

TRLs (density < 1.006 kg/L) were isolated from 4.5 mL of serum layered beneath 7 mL of 0.9% (*w*/*v*) NaCl solution (density 1.006 kg/L) in Polyallomer™ tubes (Beckman Instruments, Inc., Palo Alto, CA, USA). TRLs were separated by ultracentrifugation at 39,000 rpm for 16 h at 12 °C using an SW 41 Ti rotor in a Beckman Coulter L90K preparative ultracentrifuge (Beckman Instruments, Inc., Palo Alto, CA, USA).

TRLs were adjusted to a final volume of 1.5 mL with 0.1 M KCl. Total lipids were then extracted using the method of Folch [15]. Lipids were re-dissolved in 0.5 mL of chloroform:methanol (2:1, *v*/*v*), from which a 200 µL aliquot was taken for the separation of TGs and phospholipids (PLs) by solid-phase extraction (SPE). Normal-phase LC-Diol SPE columns (Supelclean™ LC-Diol, Supelco, Bellefonte, PA, USA) were conditioned with hexane. TGs were eluted with hexane:dichloromethane (9:1, *v*/*v*), while PLs were eluted sequentially with methanol followed by acetone (VWR Chemicals Radnor, PA, USA).

FAs from TGs and PLs were derivatized to FA methyl esters (FAMEs) as described elsewhere [16]. Two microliters of the resulting FAMEs was injected using an automatic injector (Hewlett-Packard 6890 Series, Avondale, AZ, USA) coupled to a Hewlett-Packard 5890 Series II gas chromatograph (Avondale, AZ, USA) controlled by ChemStation Rev. A-03-02 data system (Hewlett-Packard, Avondale, AZ, USA). The GC was equipped with a flame ionization detector (FID) (Hewlett-Packard, Avondale, AZ, USA) and a BPX70 SGE Analytical Science fused silica capillary column (Trajan, Victoria, Australia), 10 m long, 0.1 mm i.d., and 0.2 µm film thickness. Nitrogen was used as the carrier gas at 2.0 bar, with auxiliary air and hydrogen pressures set at 1.5 bar each. Injector and detector temperatures were held constant at 270 °C. The oven temperature program started at 190 °C, increasing at 5 °C/min to 250 °C, and then held isothermally for the remainder of the 12 min run. FAMEs were identified by comparison of retention times with those of standards injected under the same conditions. Quantification was performed by calculating the relative percentage of each FAME in relation to the total.

### 2.7. Influence of TRLs on Foam Cell Formation

In vitro experiments were conducted using the human monocytic leukemia cell line THP-1, obtained from the European Collection of Cell Cultures (Public Health England Culture Collection, Salisbury, UK). THP-1 cells were maintained in suspension at a density of (3 - 9) × 10^5^ cells/mL in RPMI 1640 medium (Biowest SAS, Nuaillé, France), supplemented with 10% (*v*/*v*) heat-inactivated fetal bovine serum, 2 mM L-alanyl-glutamine, penicillin (100 units/mL), and streptomycin (100 µg/mL). Cells were incubated at 37 °C in a humidified atmosphere containing 5% CO_2_ and 95% air. Cell viability exceeded 95% in all experiments. Differentiation into macrophages was induced by treating the cell suspension with phorbol 12-myristate 13-acetate (PMA) (Sigma-Aldrich, Poole, UK) at a final concentration of 200 ng/mL for 72 h.

Lipid uptake within THP-1 macrophages following incubation with TRLs was assessed histologically using Oil Red O staining. PMA-treated THP-1 cells were cultured at a density of 8 × 10^5^ cells/mL. The appropriate volume of TRLs was added to achieve a final concentration of 25 µg apo B, which was assessed by inmunoturbidimetry (BioSystems, Barcelona, Spain), and total protein was determined using the method of Bradford [17]. After 48 h of incubation, cells were treated with 1 mL of 60% isopropanol in water (*v*/*v*) and 1 mL of Oil Red O solution (0.2% *w/v* in 40% isopropanol in water, *v*/*v*) for 10 min. Cells were visualized using an inverted Motic AE21 Series microscope (Barcelona, Spain) equipped with a Moticam 2500 5.0 M Pixel Live Resolution camera (Motic, Barcelona, Spain), and images of the wells were captured at 40× magnification.

Cells were lysed by sonication for 5 s (twice) at 50 W using a Bandelin Sonopuls HD2070 sonicator (Bandelin electronic, Berlin, Germany). Cell lysates were transferred to a glass tube for lipid extraction, separated into TGs and PLs, followed by FA methylation and GC analysis, according to the procedure described above.

PMA-treated THP-1 monocytes were seeded in 6-well plates at a cell density of 8 × 10^5^ cells/mL. After differentiation into macrophages, the appropriate volume of TRLs was added to reach a final concentration of 25 µg apoB, and cells were incubated for 48 h. Total RNA was extracted from the cells using TRIsure™ reagent, following the manufacturer’s instructions (Bioline, London, UK). The concentration and purity of the extracted mRNA were determined by absorbance at 260 nm and 280 nm directly using a Nanodrop Lite spectrophotometer (Thermo Scientific, Waltham, MA, USA). The relative expression levels of target genes were determined by quantitative real-time polymerase chain reaction (qPCR). For this purpose, mRNA was reverse transcribed into complementary DNA (cDNA), which was then used as a template for PCR amplification of each gene analyzed, using the iScript™ Reverse Transcription Supermix for RT-qPCR (Bio-Rad, Hercules, CA, USA) in a thermal cycler (C1000™ Thermal Cycler, Bio-Rad, CA, USA), and the following protocol was applied: 5 min at 25 °C for primer annealing; 30 min at 42 °C for cDNA synthesis; and 5 min at 85 °C for enzyme inactivation. After completion, the samples were immediately placed on ice. Each RT reaction batch included a negative control without RNA to ensure reagent purity and to rule out possible genomic DNA contamination in the RNA samples.

Primer sequences were designed using Primer Premier 5.0 software (Premier Biosoft International, Palo Alto, CA, USA). The sequences of the designed primers are shown in Supplementary Table S2 and were synthesized by Sigma-Genosys Ltd. (Haverhill, UK). The qPCR reaction was performed using Precision PLUS™ Mastermix (Primer Design, Chandler’s Ford, UK). Reactions were run on a CFX96™ Real-Time System thermal cycler (Bio-Rad, CA, USA). PCR products were analyzed using Opticon Monitor™ Analysis Software V 2.01 (MJ Research Inc., Waltham, MA, USA). Relative quantification of amplified cDNA was normalized against the expression of the glyceraldehyde-3-phosphate dehydrogenase (GAPDH) gene. Relative gene expression changes were calculated using the 2^(−ΔΔCt)^ method.

### 2.8. Statistical Analysis

Results are presented as mean ± standard deviation expressed as absolute values or as changes from baseline.

Statistical significance of postprandial changes in the assays performed between obese and normal-weight adults was analyzed using the unpaired Student’s *t*-test after assessing for normality using the Kolmogorov–Smirnoff method. For adiponectin, leptin, MCP-1, SRB1, LRP1, CD36, SRA2 and LDLR, normality was not reached, so the non-parametric Wilcoxon Signed-Rank test was employed. Statistical significance of baseline and postprandial concentrations of inflammation markers between normal-weight and obese groups of adolescents and adults was assessed by two-way ANOVA followed by multiple comparisons with Bonferroni’s test. Bonferroni correction, chosen for its strong control of Type I error, was applied to the multi-group analyses of cellular fatty acid species. For two-group comparisons of distinct variables, standard pairwise tests were used without correction to balance the risks of Type I and Type II error. Differences were considered statistically significant at *p* < 0.05. Data analysis was performed using SPSS Statistics software, v. 29 (IBM Corp., Armonk, NY, USA).

## 3. Results

### 3.1. Characteristics of Study Population

The biochemical characteristics of the participants are presented in Table 2. Compared to the normal-weight group, obese individuals exhibited significantly higher body weight, BMI, systolic blood pressure, fasting glucose, fasting insulin, Homeostatic Model Assessment of Insulin Resistance (HOMA-IR), and TG, total cholesterol, and Low-Density Lipoprotein Cholesterol (LDL-c) concentrations in serum. Conversely, High-Density Lipoprotein Cholesterol (HDL-c) concentrations were significantly lower in the obese group. There were no significant differences in the remaining parameters between groups.

### 3.2. Postprandial Adipokines and Inflammation-Related Parameters

**Figure 2:**
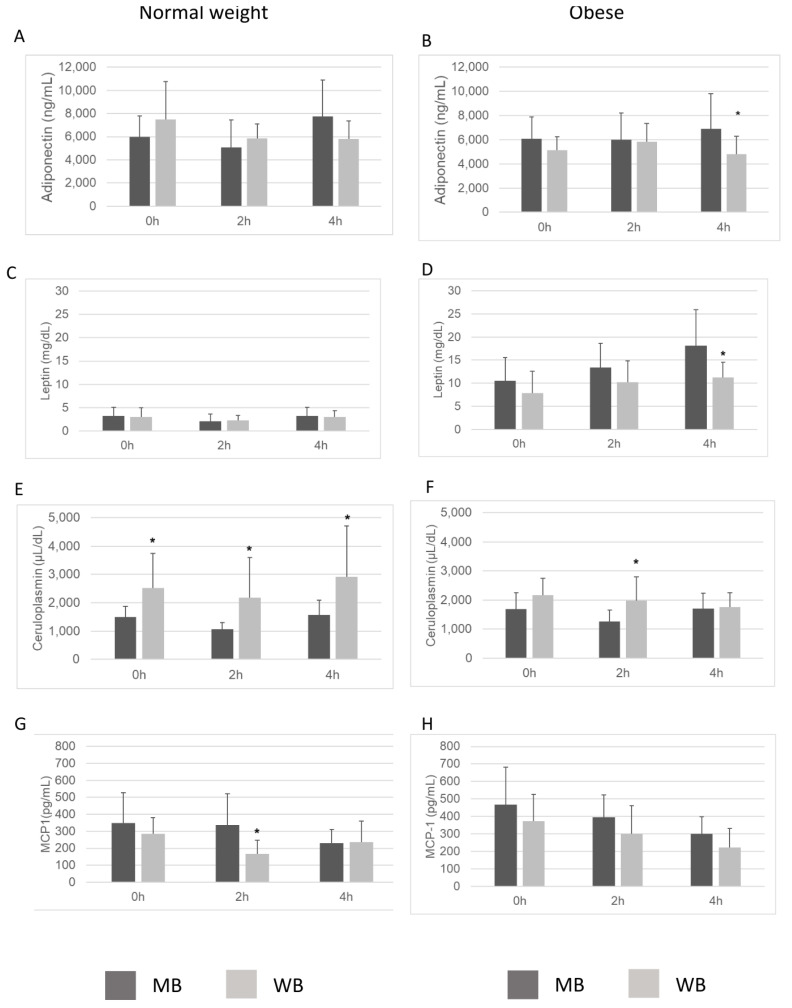
Postprandial changes in serum concentration of adipokines and inflammation-related parameters: Adiponectin (**A**,**B**), Leptin (**C**,**D**), Ceruloplasmin (**E**,**F**), and MCP-1 (**G**,**H**) in obese and normal-weight individuals after the intake of the Mediterranean-style breakfast (MB) and the Western-style breakfast (WB) at 2 h and 4 h postprandially. * *p* < 0.05, vs. MB.

Figure 2 illustrates the postprandial changes in serum concentrations of adipokines and inflammation-related parameters at 2 h and 4 h after the consumption of either the MB or the WB in normal-weight and obese individuals relative to baseline. Regarding adiponectin concentrations, no significant differences were found between the MB and the WB at any time in normal-weight subjects (Figure 2A). However, in obese individuals, adiponectin concentration was significantly higher 4 h after consumption of the MB (Figure 2B). Similarly, leptin concentration did not show significant differences in blood plasma between the two experimental meals at any time in normal-weight subjects (Figure 2C). However, in obese individuals, lower leptin concentrations were observed at 4 h postprandially after the WB (*p* < 0.05) (Figure 2D). A trend towards an increase in serum leptin concentration was observed following the consumption of the MB, and was particularly evident at 4 h postprandially in both groups. Markedly higher concentrations of ceruloplasmin were observed at both 2 h and 4 h postprandially in normal-weight individuals who consumed the WB compared to the MB (*p* < 0.05) (Figure 2E). A similar trend was observed in obese individuals, although significant differences were only detected at 2 h postprandially (Figure 2F). Regarding MCP-1, we found higher concentrations at 2 h postprandially in normal-weight individuals after consuming the MB compared to the WB (Figure 2G). Although a similar trend toward higher MCP-1 levels was observed in samples derived from the MB, no significant differences were detected at the remaining time points (Figure 2H). A decreasing trend in serum MCP-1 was observed in both groups, regardless of the type of breakfast consumed.

### 3.3. Fatty Acid Composition of Triglycerides in Triglyceride-Rich Lipoproteins

The FA composition of TGs extracted from TRLs from normal-weight and obese individuals is displayed in Table 3. In normal-weight individuals, before the MB or the WB, the FA composition of TRL particles was similar and differed in gondoic acid (20:1 *n*-9) (*p* < 0.05) only, although its concentration was negligible. At 2 h postprandially, the different lipid sources were evident in TRL particle composition and were reflected in the total SFA and MUFA content. The presence of SFAs was significantly higher in the TRLs from individuals who consumed the WB, mainly due to the elevation of myristic (14:0) and palmitic (16:0) acids. Likewise, MUFAs were particularly elevated in those derived from the MB (*p* < 0.001), with oleic acid (18:1 *n*-9) being the main contributor (*p* < 0.01). At 4 h, the myristic and palmitic acid content continued to be much higher in TRLs isolated after consuming the WB (*p* < 0.001 in both cases), together with stearic acid (18:0). The total MUFA content at 4 h remained significantly higher in TRLs derived from the MB compared to the WB. Again, this was due to the higher content of oleic acid, but also of palmitoleic (16:1 *n*-7) and gondoic acids.

In obese individuals, both experimental breakfasts affected TRL SFAs and MUFAs similarly to the normal-weight group at 2 h postprandially. SFAs were significantly higher after WB, driven by increases in myristic, palmitic, and stearic acids. MUFAs also differed, with oleic acid substantially elevated after MB (*p* < 0.01). At 4 h, SFA differences persisted: WB further increased myristic, palmitic, and stearic acids, whereas MB did not alter SFAs from baseline. Conversely, MUFAs remained higher in TRLs after the MB. No significant differences were observed between breakfasts in the postprandial period for the total PUFA content of TRLs. However, it was observed that the MB resulted in a greater increase in α-linolenic (18:3 *n*-3) acid compared to the WB (*p* < 0.05), while concentrations of arachidonic acid (20:4 *n*-6) in the particles were lower 4 h after consuming the WB (*p* < 0.001).

### 3.4. Fatty Acid Composition of Phospholipids in Triglyceride-Rich Lipoproteins

The FA composition of PLs in TRL particles from normal-weight and obese individuals is presented in Table 4. In normal-weight individuals, the lipid content of breakfasts influenced the FA composition of PLs very slightly. In fact, at 2 h postprandially, differences were only significant for PUFAs *n*-6, due to the higher linoleic acid (18:2 *n*-6) content after the WB (*p* < 0.05). Likewise, no significant differences were observed at 4 h postprandially, although certain individual SFAs displayed significant variability, such as myristic, stearic and arachidic (20:0) acids.

In obese individuals, at 2 h postprandially, α-linolenic and gondoic acids were elevated after the MB, though total FA content remained similar between meals. In contrast, by 4 h postprandially, the lipid composition of the breakfasts had a more pronounced effect on PL profiles. The WB elevated total SFA content compared to the MB (*p* < 0.05), due to the contribution of stearic acid, despite a decline in myristic acid. The MB also influenced the PL-FA profile, resulting in significantly higher MUFA levels (*p* < 0.05). The most notable increases were observed in palmitoleic and oleic acids (*p* < 0.05).

### 3.5. Influence of Experimental Breakfasts on Lipid Accumulation in THP-1 Macrophages

**Figure 3:**
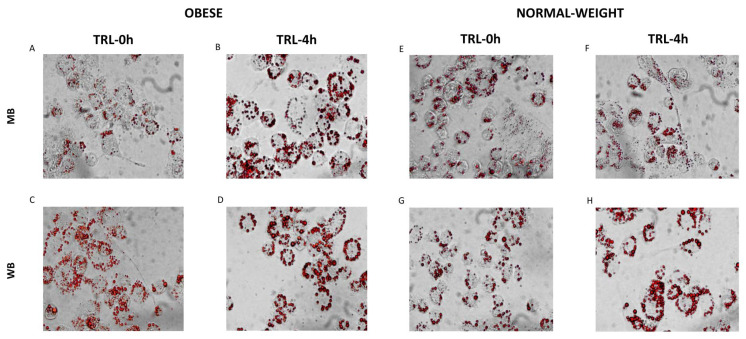
Intracellular lipid accumulation in THP-1 macrophages after postprandial TRL exposure in obese and normal-weight subjects after the intake of the Mediterranean-style breakfast (MB) and the Western-style breakfast (WB) at baseline (0 h), (**A**,**C**,**E**,**G**) and 4 h (**B**,**D**,**F**,**H**) postprandially.

After incubation of THP-1 macrophages with postprandial TRLs, the intracellular lipid accumulation was visualized by staining with Oil Red O (Figure 3). A substantial increase in intracellular lipids was observed following incubation with TRLs obtained from obese subjects 4 h after the intake of both meals, as compared to cells treated with baseline TRLs (Figure 3A–D). However, when using 4 h TRLs isolated from normal-weight individuals, increased lipid accumulation was evident only when the WB was consumed (Figure 3E–H).

### 3.6. Fatty Acid Composition of Triglycerides in Macrophages Treated with TRLs

Incubation of THP-1 macrophages with postprandial TRL particles led to marked changes in intracellular FA composition compared to untreated controls. With TRLs obtained at 2 h postprandially from normal-weight subjects, changes were regarded to myristic and palmitoleic acids only (Table 5). However, at 4 h postprandially, TRLs from the MB significantly reduced SFAs compared to the control (*p* < 0.001). Both breakfasts led to a significant increase in intracellular *n*-6 PUFAs, particularly after the MB, driven by elevated linoleic acid.

Intracellular FA concentrations in macrophages incubated with TRLs from obese individuals showed greater variation than those from normal-weight individuals (Table 6), although at 2 h postprandially, no alterations of total SFAs, MUFAs, or PUFAs were found. In contrast, at 4 h postprandially, TRLs from both breakfasts reduced intracellular SFA levels (*p* < 0.01) and increased *n*-6 PUFA concentrations (*p* < 0.001) compared to control cells. More importantly, TRLs from the WB led to higher intracellular SFAs, and lower MUFAs and arachidonic acid levels (*p* < 0.01) compared to the MB.

### 3.7. Modulation of the Gene Expression of Cellular Receptors

**Figure 4:**
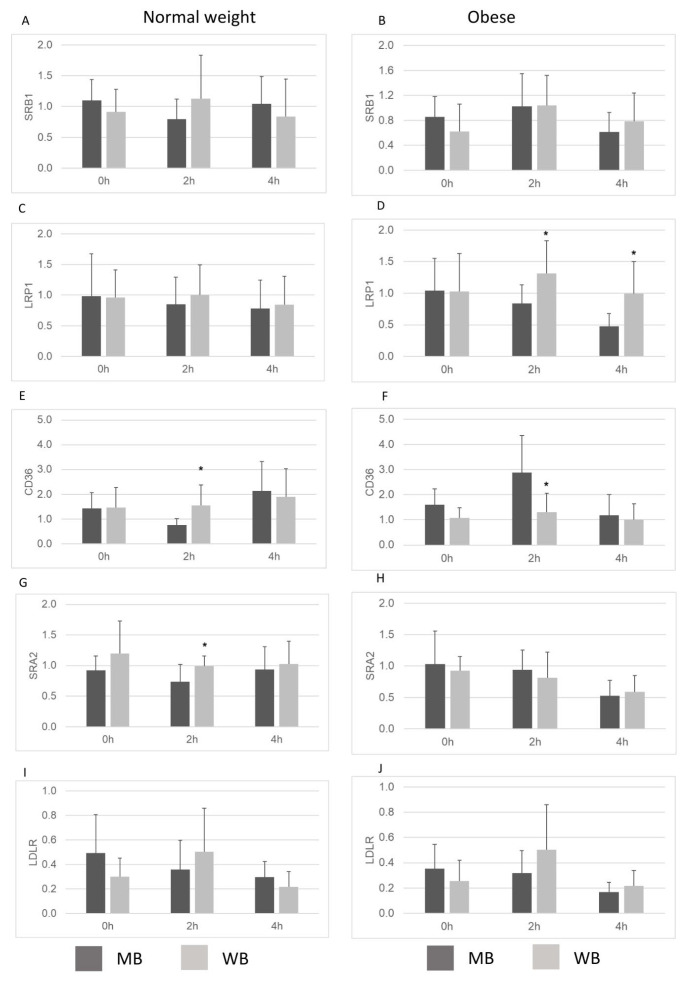
Gene expression of membrane receptors Scavenger Receptor Class B Type 1 (SRB1), (**A**,**B**), Low-Density Lipoprotein Receptor-Related Protein 1 (LRP1), (**C**,**D**), Cluster of Differentiation 36 (CD36), (**E**,**F**), Scavenger Receptor Class A Member 2 (SRA2, (**G**,**H**), and Low-Density Lipoprotein Receptor (LDLR), (**I**,**J**), related to lipoprotein uptake in macrophages incubated with TRLs in normal-weight and obese subjects after the intake of the Mediterranean-style breakfast (MB) and the Western-style breakfast (WB) at 2 h and 4 h postprandially. * *p* < 0.05, vs. MB.

Figure 4 shows the gene expression of membrane receptors related to lipoprotein uptake in macrophages incubated with TRLs. At 2 h postprandially, no significant differences were observed in Scavenger Receptor Class B Type 1 (SRB1) (Figure 4A,B) or Low-Density Lipoprotein Receptor (LDLR) (Figure 4I,J) expression regardless of breakfast type or group. However, Low-Density Lipoprotein Receptor-Related Protein 1 (LRP1) expression was lower in obese subjects after the MB compared to the WB (*p* < 0.05) (Figure 4D). Regarding Cluster of Differentiation 36 (CD36), TRLs from normal-weight individuals who consumed the WB induced higher gene expression compared to those from the MB (*p* < 0.05) (Figure 4E). In contrast, obese individuals showed lower CD36 expression after the WB (*p* < 0.05) (Figure 4F). Similarly, Scavenger Receptor Class A Member 2 (SRA2) expression was significantly elevated in macrophages treated with TRLs from normal-weight individuals who consumed the WB, in comparison with the MB (*p* < 0.05) (Figure 4G), but no such difference was found in macrophages exposed to TRLs from obese subjects (Figure 4H). At 4 h postprandially, no significant differences were found in SRB1 and LDLR expression between MB and WB in either group. For LRP1, a lower expression was again observed in obese individuals following MB compared to the WB. No differences in CD36 and SRA2 expression were observed at 4 h between breakfasts in either group.

## 4. Discussion

Obesity is recognized as a global health priority and is closely associated with a range of chronic diseases, including cardiovascular disease, metabolic syndrome, type 2 diabetes, and certain types of cancer [18]. The pathophysiology of obesity involves metabolic dysregulation, oxidative stress, and a state of chronic low-grade inflammation. White adipose tissue, functioning as an endocrine organ, plays a central role in this inflammatory milieu by secreting pro-inflammatory cytokines that impair insulin sensitivity [19]. In this context, increasing attention has been directed toward postprandial hypertriglyceridemia and its contribution to atherosclerosis, particularly through the atherogenic potential of TRL [20,21,22], whose composition and metabolic fate may differ significantly between individuals with obesity and those of normal weight and may have an influence on the inflammatory process.

Ceruloplasmin has recently gained attention as a minor acute-phase reactant mediated by cytokines that increases during low-grade inflammation and as a useful biomarker to identify patients with a high risk of cardiovascular disease [23]. However, postprandial responses of this protein remain insufficiently characterized. In the present study, the MB significantly reduced serum ceruloplasmin concentrations compared to the WB at 2 h and 4 h postprandially in normal-weight individuals, and at 2 h in obese individuals. This finding aligns with our previous observations in adolescents [16], where ceruloplasmin was higher in individuals with obesity and showed limited variation during the postprandial period. These results indicate that ceruloplasmin concentrations were sensitive to the quality of dietary fat consumed. In alloxan-induced diabetic rats, pretreatment with arachidonic acid restored plasma ceruloplasmin concentrations, an effect not observed with stearic or oleic acid, preventing pancreatic β cell damage. These mechanisms could be explained by its ability to revert the altered concentrations of ceruloplasmin induced by alloxan [24]. Notably, our current results suggest that ceruloplasmin fluctuations are meal-dependent, with the WB inducing an increase, particularly in normal-weight subjects.

Macrophages produce significant amounts of MCP-1 in response to various pro-inflammatory stimuli and tissue injuries [25] as a key factor in attracting monocytes, which then differentiate into macrophages at the injury site, creating a feedback loop. Several studies have found no difference in circulating MCP-1 levels between lean and obese adults [26], although MCP-1 has also been reported to be more expressed in visceral fat than in subcutaneous fat in obese individuals compared to lean subjects [27]. In our study, fasting serum MCP-1 levels in obese individuals were approximately 30–35% higher than in normal-weight subjects. In both groups, MCP-1 concentrations decreased progressively after meal consumption, irrespective of breakfast type. At 2 h, MCP-1 levels were lower after the WB than after the MB in normal weight individuals, although this difference was not maintained at 4 h. These results suggest a modest and transient meal-dependent effect.

Although not statistically analyzed, we also observed that serum leptin concentration, a key adipokine involved in energy homeostasis and inflammation, was higher at baseline in individuals with obesity compared to the normal-weight group, reflecting leptin resistance. This condition, characterized by a diminished responsiveness of target tissues to leptin, impairs the regulation of hunger and energy balance [28]. Throughout the postprandial period, leptin levels remained consistently elevated in the obese group, regardless of the type of breakfast. Conversely, as expected, plasma concentrations of adiponectin, which is also involved in metabolic regulation and inflammation, were higher in normal-weight individuals than in obese ones at baseline [29]. In the fasting state, higher concentrations of leptin have been directly or indirectly associated with CVD [30], while adiponectin has shown protective and anti-atherogenic roles, opposing hyperglycemia, inflammation, lipotoxic damage, and insulin resistance. In the present study, postprandial concentrations of both hormones did not differ between breakfasts in normal-weight individuals, while in obese individuals, the WB induced a reduction in adiponectin at 4 h. Taken together, these results suggest that the postprandial hormonal response was largely stable and only minimally modified by breakfast type.

Evidence regarding postprandial leptin and adiponectin secretion remains inconsistent [31]. Some studies report no changes, as well as increased postprandial leptin, in normal-weight subjects [32,33]. For adiponectin, both increased and unchanged levels have been reported in normal-weight and obese individuals. According to English et al. [34], plasma adiponectin increases postprandially in obese but not in normal-weight subjects. Paniagua et al. [5] conducted a postprandial trial with three diets rich in SFAs, MUFAs, or carbohydrates, respectively, finding that adiponectin plasma concentrations did not differ significantly among insulin-resistant participants. On the other hand, Lozano et al. [35] also conducted a postprandial trial in healthy young adults who received meals enriched in olive oil, walnuts or butter. Postprandially, higher concentrations of adiponectin were displayed after the consumption of the walnut-enriched meal, with similar concentrations of adiponectin between the olive oil and butter-enriched meals. In contrast, leptin concentrations were higher in walnut- and olive oil-enriched meals with lower concentrations after the butter-enriched meal, similarly to our results. Therefore, the lack of consensus due to differences in diet type, nutritional status, and other factors highlights the complexity of hormonal regulation after meals and underscores the need for further research to clarify these mechanisms.

The assessment of the FA composition of TGs in TRLs revealed a strong influence of the dietary fat source. At 2 h postprandially, TRL obtained from individuals after consumption of the WB contained higher concentrations of SFAs, especially myristic and palmitic acids, and lower concentrations of MUFAs, particularly oleic acid, both in the normal-weight and obese groups. These differences persisted and became more marked at 4 h, reflecting the direct incorporation of dietary FAs into postprandial TRLs [36]. Their clinical relevance is important, as the predominance of SFAs in TRLs predisposes to greater formation of VLDLs and consequently higher LDL concentrations, with their associated cardiovascular risk [37]. Additionally, SFAs negatively affect insulin signaling and glucose metabolism [38,39] and contribute to fat accumulation in the liver [40], which are key features of metabolic impairment.

In contrast, we did not find such notable differences in the FA composition of PLs in TRLs after either experimental meal in either group of individuals, as there were no significant differences in total SFAs, MUFAs or PUFAs. As in our previous study [16], PL composition showed limited responsiveness to the acute dietary intervention, consistent with the predominant contribution of endogenous FAs to this lipid fraction [41,42]. While TG composition in CMs reflects the composition of dietary FAs, PLs are only partially derived from the dietary fats, with an important participation of endogenous FAs [43]. The CM assembly initiates when a nascent apoB-48 is co-translationally inserted into the endoplasmic reticulum (ER). The microsomal TG transfer protein (MTP) facilitates the transfer of TGs and PLs from the ER membrane and cytosolic lipid droplets to the growing lipoprotein particles. Therefore, PLs incorporated into CMs are largely derived from the endogenous ER membrane pool, which contributes to the relatively stable FA composition of these lipid fractions.

As mentioned before, postprandial TRLs include CMs, VLDLs and their remnants. CMs are secreted by enterocytes and their serum concentration peaks between 2 and 4 h after dietary fat intake [44]. On the other hand, VLDLs are produced and secreted by the liver, carrying dietary TGs but also endogenous FAs, and their concentrations increase after the consumption of fats or carbohydrates, reaching the highest around 4–6 h [45]. These particles are progressively cleared from circulation by the action of lipoprotein lipase (LPL). Therefore, the 4 h postprandial point captured TRLs containing both CMs and VLDLs at the highest concentrations, providing a balanced view of postprandial lipid metabolism. In this regard, a previous work by our group had already shown that TRLs obtained 4 h after the consumption of a high-fat meal caused the greatest accumulation of TGs in THP-1 macrophages, with a significant increase in the mRNA expression of scavenger receptors, thus inducing foam cell formation [46].

In this study, Oil Red-O staining was used to visualize the presence of lipids in THP-1 macrophages following their incubation with human TRLs (Figure 2). Lipid droplet accumulation was maximal in macrophages treated with 4 h TRLs from both groups of participants, but with a higher intensity in cells treated with TRLs from individuals with obesity. This pattern is consistent with the higher lipid content of TRLs in participants with obesity observed previously [16,47]. In addition, lipid accumulation was also higher in macrophages incubated with TRLs derived from the WB, which is consistent with the higher presence of SFAs. In this regard, Perez-Martínez et al. [48] revealed that consumption of an olive oil-rich meal leads to the formation of fewer TRL particles compared with a meal based on butter. Likewise, Botham et al. [49] showed that TRLs rich in SFAs, rather than MUFAs or PUFAs, are hydrolyzed in vitro more slowly by LPL. This suggests that enrichment of TRLs with SFAs contributes to delayed clearance from the blood, and increases the opportunity to penetrate to the subendothelial space and to interact with macrophages. The predominance of SFAs makes TGs in TRLs more compact, packed more tightly, structurally more rigid, and denser, which could facilitate a better recognition by macrophage receptors, enhancing lipid accumulation and foam cell formation [50]. Notably, the greater lipid loading observed in macrophages incubated with TRLs from obese individuals after the MB does not appear to be driven by SFAs. Obesity remodels postprandial lipoprotein metabolism, yielding TRLs with higher cholesteryl ester and TG content, larger particle size, and greater susceptibility to lipolysis, which releases more free FAs that facilitate uptake via scavenger receptors, such as CD36 [51]. In parallel, obesity-derived TRLs can activate lipid-handling programs (e.g., Trem2-associated pathways in lipid-associated macrophages) and are accompanied by reduced cholesterol efflux, thereby promoting intracellular storage independently of receptor abundance per se [52]. Moreover, chronic obesity favors apoE-poor remnants that evade hepatic clearance and accumulate in macrophage-rich niches, whereas TRLs from lean subjects remain more stable postprandially, with balanced lipid profiles that limit foam cell formation [53].

Incubation of THP-1 macrophages with TRLs obtained from individuals with obesity after the WB increased SFA concentrations in macrophages compared to the MB. The 48 h incubation of macrophages with a fixed apo B concentration of TRLs was designed to model sustained exposure to postprandial remnants, as occurs in states of chronic postprandial lipemia, rather than the transient peak following a single meal. This experimental ‘stress test’ elicits robust lipid uptake and gene expression changes relevant to atherogenic pathways. The observed lipid accumulation demonstrates the capacity of TRLs to directly induce a foam cell-like phenotype, a key mechanism in early plaque development. In this regard, Taskinen et al. [54] reported in vivo evidence that the magnitude and duration of remnant exposure are modifiable and clinically relevant.

Lipid accumulation by macrophages is a consequence of the different pathways for TRL uptake. Smaller TRLs, such as remnants of CMs and VLDLs, can cross the endothelial layer by active transcytosis and accumulate in the subendothelial space of blood vessels, whereas unhydrolyzed CMs and very large VLDLs are excluded [55]. By expressing LPL, macrophages convert large TRLs into remnants, hydrolyzing TGs from TRLs into free FAs that are internalized and re-esterified intracellularly. Furthermore, TGs can also be internalized by the uptake of complete TRL particles through receptor-mediated pathways [47]. In addition to FA uptake, these receptors are also involved in the internalization of oxidized lipoproteins, leading to increased intracellular lipid accumulation and foam cell formation [56]. Several studies revealed the beneficial effects on plasma lipid metabolites of the replacement of dietary SFAs with MUFAs when comparing diets with FA sources of different degrees of unsaturation [57,58]. However, other studies did not find large differences in lipid parameters when comparing butter- and coconut oil-enriched diets versus an olive oil-enriched diet [59]; palm olein versus olive oil [60]; and butter, coconut oil, olive oil, and canola oil postprandially [61]. The present results specifically highlight the impact of SFA-rich TRLs on macrophage lipid accumulation.

The increased SFA intracellular content observed after treatment with TRLs obtained from obese individuals after WB was concomitant with increased CD36 and LRP1 gene expression with 2 h TRLs and decreased with 4 h TRLs. These differences, though small, varied between time points, suggesting that receptor expression may be influenced by multiple factors beyond TRL composition. The current results might reflect postprandial lipid metabolism dysfunctions in individuals with obesity, contributing to atherogenic processes [62]. In this line, Nakata et al. [63] reported that CD36 expression in macrophages was concomitantly suppressed after the cell extravasation into the aortic wall. Moreover, it has been reported that the expression of CD36 in foam cells can be repressed by cytokines, especially interferon-gamma [64], whose production is stimulated by oxLDL [65]. Conversely, Mueller et al. [66] showed an elevated LRP1 expression in progressing atherosclerotic plaques, compared to those undergoing regression, in a murine model of atherosclerosis. Nevertheless, despite CD36 and LRP1 being major gateways for TRL uptake, lipid accumulation does not always scale with receptor expression. In non-obese subjects, lipid-laden macrophages may emerge without proportional CD36/LRP-1 upregulation, possibly due to balanced lipid metabolism or protective subpopulations like lipid-associated macrophages (LAMs) [67]. These LAMs show high lipid activity but low inflammation, decoupling accumulation from receptor levels alone. This mismatch suggests compensatory mechanisms, such as FA transport genes or stress responses, sustain lipid storage without receptor overexpression in lean states [68].

Therefore, our findings suggest that dietary fat quality influenced TRL FA composition and macrophage lipid uptake, and that these effects differed between normal-weight and obese individuals. A key strength is that the findings come from a randomized controlled trial conducted in the postprandial state, involving normal-weight and obese adults who consumed either an MB or a WB. We found that WB consumption triggers a more pro-inflammatory and pro-atherogenic response than MB. The FA composition in TRLs was significantly affected by dietary fat source, with implications for cardiovascular risk, especially in obese individuals. TRLs from a WB had higher SFA levels due to butter, while those from an MB were rich in MUFAs from olive oil. Moreover, WB-derived TRLs promoted greater lipid accumulation in macrophages and upregulated scavenger receptors CD36 and LRP1, contributing to foam cell formation and increased cardiovascular risk. However, this study has several limitations that should be considered when interpreting the findings. The sample size, though appropriate for a mechanistic crossover trial, inherently limits its statistical power and precision, particularly concerning secondary outcomes like gene expression and fatty acid fractions. Furthermore, the demographic homogeneity of our participant pool, comprising only adult men aged 20–40 years, means the observed effects of the dietary interventions on postprandial TRL atherogenicity cannot be assumed to translate to other critical groups. Additionally, recruitment from a single geographic location (Seville, Spain) introduces potential cultural and dietary biases. Finally, the Bonferroni correction was applied solely to the multi-group comparisons of cellular fatty acids. While this controls Type I error for those specific findings, the uncorrected pairwise comparisons throughout the study carry an inherent collective risk of false positives when interpreted globally.

## 5. Conclusions

In conclusion, this study underscores the critical role of dietary fat quality in modulating postprandial metabolic and inflammatory responses. The consumption of an MB, with olive oil as the primary fat source, appears to confer protective effects by attenuating lipid accumulation in macrophages and reducing inflammation (i.e., ceruloplasmin) and metabolic disturbances (i.e., TRL and intracellular fatty acid composition), compared to a WB. However, these findings should be interpreted with caution, given the design and sample size of the study, and may not be generalizable to all populations or dietary contexts. They support the potential value of dietary strategies focused on fat quality to reduce cardiovascular risk, but further research in diverse cohorts is needed to confirm these effects. Furthermore, the differential expression of lipid uptake receptors highlights the complexity of postprandial lipid signaling and suggests the need for personalized nutritional approaches. Future human studies are warranted to further elucidate the mechanisms underlying receptor regulation and lipid metabolism during the postprandial phase.

## Figures and Tables

**Table 1:** Fatty acid composition of olive oil and butter used as the main fat source in both breakfasts ^a^.

Fatty Acid (%)	Olive Oil	Butter
10:0	–	1.77 ± 0.03
12:0	–	2.66 ± 0.03
14:0	–	10.15 ± 0.04
14:1 *n*-5	–	1.13 ± 0.01
16:0	9.94 ± 0.02	33.18 ± 0.17
16:1 *n*-7	0.81 ± 0.00	2.33 ± 0.04
18:0	3.31 ± 0.01	13.85 ± 0.07
18:1 *n*-9t	–	3.11 ± 0.13
18:1 *n*-9c	77.69 ± 0.05	27.47 ± 0.11
18:2 *n*-6	6.34 ± 0.02	3.47 ± 0.03
18:3 *n*-3	1.14 ± 0.01	0.56 ± 0.02
20:0	0.45 ± 0.02	–
20:1 *n*-9	0.32 ± 0.01	0.31 ± 0.01

^a^ Data are presented as mean ± SD.

**Table 2:** Characteristics of the study participants ^a^.

	Normal-Weight	Obese
N	12	12
Age	27.3 ± 3.0	35.8 ± 12.0 *
Weight (kg)	74.9 ± 7.8	112.9 ± 13.5 ***
Height (cm)	179.6 ± 7.0	180.8 ± 5.2
BMI (Kg/m^2^)	23.2 ± 1.1	34.7 ± 5.0 ***
SBP (mmHg)	110.9 ± 12.7	130.8 ± 16.3 **
DBP (mmHg)	68.9 ± 7.3	74.5 ± 10.1
Fasting glucose (mg/dL)	73.7 ± 4.7	83.6 ± 8.3 **
Fasting insulin (μU/mL)	4.9 ± 2.7	12.6 ± 3.5 ***
HOMA-IR	0.9 ± 0.5	2.4 ± 1.2 ***
Triglycerides (mg/dL)	78.7 ± 24.5	129.7 ± 60.3 *
Cholesterol (mg/dL)	172.5 ± 32.6	202.9 ± 34.6 *
LDL-c (mg/dL)	94.7 ± 19.4	158.7 ± 16.7 ***
HDL-c (mg/dL)	57.5 ± 9.5	45.9 ± 8.5 **

^a^ Data are presented as mean ± SD; N: number of participants. BMI: body mass index; HOMA-IR: homeostatic model assessment of insulin resistance; LDL-c: low-density lipoprotein cholesterol; HDL-c: high-density lipoprotein cholesterol; SBP: systolic blood pressure; DBP: diastolic blood pressure. Statistical differences between the two groups were analyzed using an unpaired Student’s *t*-test. * *p* < 0.05, ** *p* < 0.01, *** *p* < 0.001 vs. normal-weight group.

**Table 3:** Fatty acid composition of triglycerides in triglyceride-rich lipoproteins (TRLs) from normal-weight and obese individuals ^a^.

	Normal-Weight						Obese					
Fatty Acids	0 h		2 h		4 h		0 h		2 h		4 h	
	MB	WB	MB	WB	MB	WB	MB	WB	MB	WB	MB	WB
14:0	1.5 ± 1.0	1.0 ± 0.5	1.2 ± 0.6	3.2 ± 1.1 ***	1.6 ± 0.2	3.0 ± 0.8 ***	1.3 ± 0.6	1.2 ± 0.5	1.1 ± 0.4	2.6 ± 1.0 ***	1.5 ± 0.3	2.9 ± 0.9 ***
14:1 *n*-5	0.5 ± 0.3	0.3 ± 0.2	0.2 ± 0.2	0.6 ± 0.3 **	0.5 ± 0.1	0.6 ± 0.2	0.1 ± 0.1	0.2 ± 0.1	0.3 ± 0.3	0.4 ± 0.2	0.2 ± 0.1	0.5 ± 0.1 ***
16:0	21.3 ± 3.3	20.1 ± 2.3	20.9 ± 2.1	24.0 ± 2.9 **	19.6 ± 1.5	25.3 ± 3.3 ***	21.8 ± 2.9	22.0 ± 2.7	20.8 ± 3.0	23.6 ± 2.3 *	21.2 ± 2.8	25.2 ± 2.6 **
16:1 *n*-7	4.7 ± 2.1	3.6 ± 1.2	3.9 ± 1.3	4.2 ± 1.1	3.5 ± 0.7	2.4 ± 0.6 **	3.6 ± 1.0	3.5 ± 1.0	3.9 ± 0.4	4.0 ± 0.6	3.9 ± 0.6	4.5 ± 1.1
18:0	6.3 ± 2.6	5.4 ± 2.0	6.8 ± 3.2	6.2 ± 1.1	5.8 ± 1.3	7.9 ± 2.5 *	4.3 ± 1.5	5.3 ± 2.7	4.1 ± 0.8	5.1 ± 0.9 *	4.2 ± 0.9	6.0 ± 1.6 **
18:1 *n*-9	38.6 ± 1.6	39.3 ± 4.4	43.8 ± 4.7	37.3 ± 4.1 **	44.3 ± 3.5	36.8 ± 3.3 ***	38.4 ± 3.4	35.1 ± 3.0	41.3 ± 4.3	34.4 ± 3.2 ***	40.5 ± 4.0	34.3 ± 2.7 ***
18:2 *n*-6	20.8 ± 3.6	24.2 ± 5.8	18.5 ± 3.7	19.2 ± 4.6	18.2 ± 1.8	15.5 ± 4.1	24.0 ± 5.2	26.0 ± 4.9	22.2 ± 3.5	23.6 ± 4.7	20.9 ± 2.2	21.6 ± 3.8
18:3 *n*-6	0.5 ± 0.2	0.7 ± 0.3	0.5 ± 0.3	0.5 ± 0.2	0.5 ± 0.2	2.3 ± 0.6 ***	0.8 ± 0.3	0.7 ± 0.3	0.6 ± 0.3	0.7 ± 0.2	0.8 ± 0.2	0.8 ± 0.2
18:3 *n*-3	0.8 ± 0.4	0.8 ± 0.6	0.9 ± 0.6	0.6 ± 0.3	0.6 ± 0.1	1.7 ± 0.5 ***	0.7 ± 0.3	0.7 ± 0.5	0.5 ± 0.2	0.5 ± 0.2	0.7 ± 0.2	0.5 ± 0.1 *
20:0	0.6 ± 0.6	0.4 ± 0.4	0.7 ± 0.7	0.3 ± 0.2	0.4 ± 0.1	0.2 ± 0.1 ***	0.4 ± 0.2	0.4 ± 0.3	0.4 ± 0.2	0.2 ± 0.1 *	0.4 ± 0.1	0.4 ± 0.1
20:1 *n*-9	0.9 ± 0.6	0.5 ± 0.3 *	0.5 ± 0.4	0.2 ± 0.3	0.3 ± 0.1	0.4 ± 0.1 **	0.2 ± 0.2	0.4 ± 0.4	0.3 ± 0.4	0.7 ± 0.7	0.3 ± 0.1	0.3 ± 0.1
20:2 *n*-6	0.3 ± 0.1	0.4 ± 0.1	0.3 ± 0.1	0.3 ± 0.1	0.2 ± 0.1	0.4 ± 0.1 ***	0.5 ± 0.2	0.5 ± 0.1	0.5 ± 0.1	0.5 ± 0.1	0.3 ± 0.1	0.3 ± 0.1
20:4 *n*-6	2.2 ± 0.6	2.6 ± 1.1	2.0 ± 0.6	1.9 ± 0.4	1.6 ± 0.2	1.4 ± 0.2	2.6 ± 0.5	2.6 ± 0.7	2.2 ± 0.3	2.4 ± 0.6	1.5 ± 0.2	0.4 ± 0.1 ***
20:5 *n*-3	0.6 ± 0.2	0.4 ± 0.3	0.7 ± 0.6	0.3 ± 0.3	2.2 ± 0.2	2.1 ± 0.4	0.4 ± 0.2	0.3 ± 0.1	0.4 ± 0.2	0.3 ± 0.2	2.4 ± 0.3	2.2 ± 0.6
SFA	29.2 ± 3.4	26.7 ± 2.5	27.1 ± 2.8	33.8± 4.6 ***	27.3 ± 2.2	36.5 ± 5.5 ***	27.6 ± 4.3	29.5 ± 4.3	26.4 ± 3.5	32.0 ± 2.7 ***	27.2 ± 3.2	34.7 ± 3.6 ***
MUFA	45.0 ± 1.6	43.6 ± 5.0	48.7 ± 3.3	42.3± 3.7 ***	48.6 ± 3.1	40.1 ± 3.2 ***	42.4 ± 3.4	39.6 ± 2.4	45.6 ± 3.9	40.1 ± 2.8 **	45.0 ± 3.7	39.2 ± 2.6 ***
PUFA *n*-6	23.9 ± 4.0	28.1 ± 6.2	22.1 ± 3.2	22.3 ± 4.6	20.4 ± 1.8	19.6 ± 4.2	28.2 ± 5.2	28.9 ± 3.9	25.9 ± 3.5	26.4 ± 3.6	23.5 ± 2.2	23.6 ± 3.8
PUFA *n*-3	2.0 ± 0.5	1.7 ± 1.0	2.1 ± 1.3	1.6 ± 0.8	2.8 ± 0.2	3.8 ± 0.6 ***	1.9 ± 0.7	2.1 ± 0.8	2.1 ± 0.8	1.5 ± 0.5	3.1 ± 0.3	3.0 ± 0.8

^a^ Data are presented as mg/100 mg of triglycerides. Abbreviations: MB, Mediterranean breakfast; WB, Western breakfast; SFA, saturated fatty acid; MUFA, monounsaturated fatty acid; PUFA, polyunsaturated fatty acid. Values are expressed as mean ± standard deviation. N = 12. * *p* < 0.05, ** *p* < 0.01, and *** *p* < 0.001 vs. MB (paired Student’s *t*-test).

**Table 4:** Fatty acid composition of phospholipids in triglyceride-rich lipoproteins (TRLs) from normal-weight and obese individuals ^a^.

	Normal-Weight						Obese					
Fatty Acids	0 h		2 h		4 h		0 h		2 h		4 h	
	MB	WB	MB	WB	MB	WB	MB	WB	MB	WB	MB	WB
14:0	1.6 ± 0.5	1.0 ± 0.6 *	1.4 ± 1.3	0.7 ± 0.5	2.1 ± 0.3	1.7 ± 0.5 *	1.2 ± 0.7	0.4 ± 0.1 **	1.0 ± 0.6	0.8 ± 0.5	2.0 ± 0.5	1.3 ± 0.1 ***
14:1 *n*-5	1.3 ± 1.0	0.7 ± 0.5	0.3 ± 0.2	0.7 ± 0.5	0.4 ± 0.2	0.4 ± 0.1	0.8 ± 0.8	0.4 ± 0.3	0.5 ± 0.4	0.4 ± 0.4	0.4 ± 0.1	0.4 ± 0.1 *
16:0	25.0 ± 4.9	25.1 ± 4.0	24.3 ± 2.9	24.3 ± 1.6	21.9 ± 1.3	22.6 ± 2.5	24.4 ± 3.9	25.1 ± 4.5	24.7 ± 2.2	25.0 ± 3.7	20.5 ± 2.0	21.8 ± 1.4
16:1 *n*-7	4.0 ± 2.0	3.4 ± 2.2	4.3 ± 2.9	2.9 ± 2.8	1.9 ± 0.2	2.0 ± 0.3	3.1 ± 1.9	2.3 ± 1.8	2.6 ± 1.5	2.5 ± 2.1	2.0 ± 0.2	1.7 ± 0.2 *
18:0	18.6 ± 4.8	20.4 ± 2.6	18.9 ± 1.8	17.3 ± 2.1	15.6 ± 1.5	17.0 ± 1.2 *	19.2 ± 2.3	18.8 ± 2.9	18.2 ± 3.4	18.2 ± 1.8	15.1 ± 1.7	16.9 ± 1.4 *
18:1 *n*-9	18.2 ± 6.2	16.2 ± 2.8	17.4 ± 5.8	15.7 ± 4.3	25.6 ± 1.5	24.2 ± 1.9	15.7 ± 3.4	16.2 ± 4.3	17.2 ± 6.3	14.5 ± 2.7	26.1 ± 1.8	23.9 ± 2.1 *
18:2 *n*-6	12.8 ± 3.0	15.6 ± 3.7	14.2 ± 5.2	19.3 ± 2.4 *	19.3 ± 1.4	19.0 ± 1.6	16.8 ± 2.4	18.7 ± 4.2	16.8 ± 3.6	20.0 ± 4.4	18.2 ± 1.3	18.3 ± 1.2
18:3 *n*-6	0.3 ± 0.1	0.9 ± 0.9	0.5 ± 0.3	0.4 ± 0.2	1.2 ± 0.1	1.2 ± 0.2	0.5 ± 0.3	0.6 ± 0.5	0.4 ± 0.2	0.2 ± 0.3	1.1 ± 0.1	1.2 ± 0.1
18:3 *n*-3	2.9 ± 2.1	2.4 ± 1.8	3.7 ± 2.6	0.9 ± 0.8 **	0.3 ± 0.1	0.5 ± 0.1 ***	1.6 ± 1.5	1.2 ± 1.2	1.9 ± 1.4	0.7 ± 0.9 *	0.4 ± 0.1	0.4 ± 0.1
20:0	1.7 ± 1.4	0.5 ± 0.3 *	0.4 ± 0.2	0.4 ± 0.3	0.2 ± 0.1	0.3 ± 0.1 **	0.7 ± 0.7	0.7 ± 0.6	0.8 ± 0.8	0.4 ± 0.4	0.3 ± 0.1	0.2 ± 0.1
20:1 *n*-9	2.1 ± 1.3	1,4 ± 0.7	1.0 ± 0.9	1.4 ± 0.9	1.3 ± 0.3	1.2 ± 0.2	1.2 ± 0.7	1.0 ± 0.9	1.5 ± 1.1	0.4 ± 0.3 *	1.2 ± 0.2	1.2 ± 0.2
20:2 *n*-6	2.0 ± 0.7	2.9 ± 1.4	3.6 ± 2.2	2.5 ± 0.9	2.0 ± 1.2	1.4 ± 0.2	3.1 ± 1.1	2.8 ± 1.4	2.8 ± 1.1	3.0 ± 0.8	2.1 ± 0.6	1.8 ± 0.6
20:4 *n*-6	6.9 ± 2.3	7.6 ± 2.5	7.3 ± 3.5	9.6 ± 2.6	5.5 ± 1.0	5.0 ± 1.2	8.9 ± 2.4	8.5 ± 2.8	7.9 ± 2.1	9.3 ± 1.9	7.5 ± 0.7	7.7 ± 1.2
20:5 *n*-3	1.6 ± 1.4	0.6 ± 0.4	0.9 ± 0.8	0.8 ± 0.7	1.3 ± 0.2	1.1 ± 0.1	1.1 ± 0.9	0.8 ± 0.9	1.3 ± 1.0	0.6 ± 0.5	1.5 ± 0.4	1.3 ± 0.2
SFA	45.7 ± 8.2	46.2 ± 5.2	44.6 ± 2.8	42.5 ± 2.3	39.8 ± 1.5	41.6 ± 2.7	44.6 ± 3.9	45.0 ± 5.3	44.0 ± 3.2	44.3 ± 3.8	37.9 ± 2.1	39.8 ± 1.5 *
MUFA	25.5 ± 5.7	21.5 ± 3.1	22.8 ± 6.7	20.4 ± 5.2	29.1 ± 1.5	27.9 ± 1.9	20.1 ± 4.2	19.9 ± 3.6	21.5 ± 5.0	18.2 ± 3.1	29.6 ± 1.8	27.6 ± 2.0 *
PUFA *n*-6	21.9 ± 4.8	27.2 ± 5.4 *	26.1 ± 7.7	32.2 ± 4.4 *	27.9 ± 1.4	26.6 ± 1.8	29.8 ± 5.5	30.4 ± 7.4	28.0 ± 5.3	32.3 ± 4.5	28.9 ± 1.1	29.2 ± 1.3
PUFA *n*-3	6.9 ± 4.5	5.2 ± 2.2	6.5 ± 3.0	4.9 ± 1.3	1.6 ± 0.3	1.6 ± 0.2	5.5 ± 2.5	4.7 ± 2.7	6.4 ± 2.7	5.3 ± 1.5	1.9 ± 0.4	1.7 ± 0.2

^a^ Data are presented as mg/100 mg of phospholipids. Abbreviations: MB, Mediterranean breakfast; WB, Western breakfast; SFA, saturated fatty acid; MUFA, monounsaturated fatty acid; PUFA, polyunsaturated fatty acid. Values are expressed as mean ± standard deviation. N = 12. * *p* < 0.05, ** *p* < 0.01, and *** *p* < 0.001 vs. MB (paired Student’s *t*-test).

**Table 5:** Fatty acid composition of triglycerides in macrophages treated with TRLs from normal-weight individuals ^a^.

		0 h		2 h		4 h	
Fatty Acid	Control	MB	WB	MB	WB	MB	WB
14:0	1.6 ± 0.5	1.8 ± 1.0	1.7 ± 0.6	2.1 ± 1.2	2.1 ± 0.8 #	1.9 ± 0.3	2.2 ± 0.5 ##
14:1 *n*-5	1.1 ± 0.9	0.5 ± 0.6	0.4 ± 0.4 #	0.9 ± 0.7	0.7 ± 0.5	0.6 ± 0.2	0.7 ± 0.2
16:0	27.0 ± 6.9	24.3 ± 1.7	25.2 ± 4.0	25.0 ± 3.4	24.9 ± 4.3	18.9 ± 3.7 ##	21.2 ± 2.3
16:1 *n*-7	5.1 ± 2.9	5.2 ± 1.2	5.7 ± 2.0	6.6 ± 2.6	7.9 ± 2.4 #	2.1 ± 0.4 ##	2.1 ± 0.4 ##
18:0	21.3 ± 4.5	20.5 ± 4.4	19.6 ± 5.9	20.5 ± 4.0	19.6 ± 6.5	13.8 ± 2.3 ###	14.3 ± 1.5
18:1 *n*-9	26.4 ± 6.7	28.6 ± 5.4	27.2 ± 7.2	27.8 ± 5.5	23.8 ± 4.7	31.9 ± 2.7 #	31.2 ± 2.9
18:2 *n*-6	5.5 ± 3.4	7.2 ± 3.3	8.6 ± 6.2	7.4 ± 2.1	7.0 ± 5.7	20.8 ± 2.7 ###	17.9 ± 1.1
18:3 *n*-6	2.2 ± 1.5	1.6 ± 0.5	2.1 ± 1.1	1.7 ± 1.4	1.6 ± 0.6	2.2 ± 0.5	2.7 ± 0.2 *
18:3 *n*-3	1.7 ± 1.2	1.3 ± 0.4	1.3 ± 0.8	2.0 ± 1.3	1.2 ± 0.7	1.3 ± 0.3	1.7 ± 0.3 *
20:0	1.3 ± 0.8	0.9 ± 0.5	0.7 ± 0.3	1.7 ± 1.5	2.7 ± 2.4	0.5 ± 0.1 ##	0.6 ± 0.1 #
20:1 *n*-9	1.7 ± 1.9	1.1 ± 1.0	1.7 ± 1.3	1.1 ± 0.6	2.1 ± 0.0	0.3 ± 0.1 #	0.5 ± 0.1 *#
20:2 *n*-6	0.9 ± 1.1	0.4 ± 0.2	0.4 ± 0.2	0.7 ± 0.6	0.9 ± 1.3	0.2 ± 0.1	0.3 ± 0.1
20:4 *n*-6	1.4 ± 0.7	2.3 ± 1.1 #	1.8 ± 0.9	1.5 ± 0.8	1.3 ± 0.8	2.0 ± 0.4	1.6 ± 0.3 *
20:5 *n*-3	2.1 ± 1.5	1.5 ± 1.1	1.4 ± 1.1	0.0 ± 0.5	3.0 ± 0.0	2.7 ± 0.5	2.6 ± 0.4
Others	1.4 ± 1.4	2.7 ± 0.5 #	2.6 ± 0.9 #	2.6 ± 0.7 #	3.7 ± 0.0 ###	0.8 ± 0.2	0.8 ± 0.2
SFA	50.9 ± 9.6	47.5 ± 5.6	47.1 ± 9.0	48.9 ± 5.9	45.4 ± 7.8	35.0 ± 4.0 ###	38.3 ± 2.6
MUFA	34.1 ± 6.5	35.0 ± 5.3	34.5 ± 6.1	36.1 ± 4.9	35.3 ± 4.4	34.9 ± 2.5	34.3 ± 2.9
PUFA *n*-6	9.8 ± 3.5	11.2 ± 2.9	13.6 ± 6.4	11.4 ± 2.2	12.2 ± 5.7	25.3 ± 3.0 ###	22.4 ± 1.1 ##
PUFA *n*-3	3.7 ± 2.4	3.2 ± 1.4	3.5 ± 1.9	4.2 ± 2.5	4.5 ± 2.5	4.0 ± 0.6	4.2 ± 0.4

^a^ Data are presented as mg/100 mg of triglycerides. Abbreviations: MB, Mediterranean breakfast; WB, Western breakfast.; SFA, saturated fatty acid; MUFA, monounsaturated fatty acid; PUFA, polyunsaturated fatty acid. Values are expressed as mean ± standard deviation. N = 12. * *p* < 0.05 vs. MB; # *p* < 0.05, ## *p* < 0.01, ### *p* < 0.001 vs. control. *p*-values are Bonferroni-corrected for the multiple comparisons.

**Table 6:** Fatty acid composition of triglycerides in macrophages treated with TRLs from obese individuals ^a^.

		0 h		2 h		4 h	
Fatty Acid (%)	Control	MB	WB	MB	WB	MB	WB
14:0	1.6 ± 0.5	2.0 ± 1.4	1.6 ± 0.8	2.2 ± 1.4	1.8 ± 0.9	1.4 ± 0.1	1.8 ± 0.2 ***
14:1 *n*-5	1.1 ± 0.9	0.5 ± 0.5	0.4 ± 0.1 #	0.7 ± 0.6	0.9 ± 1.2	0.5 ± 0.1	0.6 ± 0.1 *
16:0	27.0 ± 6.9	23.2 ± 4.2	23.6 ± 3.2	23.3 ± 3.7	22.8 ± 5.5	20.9 ± 1.7 #	22.6 ± 2.8
16:1 *n*-7	5.1 ± 2.9	6.9 ± 3.1	4.6 ± 2.3	6.6 ± 2.2	5.6 ± 2.5	2.3 ± 0.5 ##	2.4 ± 0.6 ##
18:0	21.3 ± 4.5	15.0 ± 6.2 #	16.1 ± 2.9 ##	19.0 ± 6.0	21.5 ± 4.8	14.3 ± 1.4 ###	15.4 ± 2.3 ##
18:1 *n*-9	26.4 ± 6.7	28.9 ± 7.5	30.6 ± 4.3	30.1 ± 9.4	24.9 ± 4.7	29.6 ± 1.6	27.3 ± 2.0 **
18:2 *n*-6	5.5 ± 3.4	12.1 ± 6.8 #	10.7 ± 3.0 ##	6.6 ± 3.2	7.8 ± 4.9	20.9 ± 1.4 ###	20.6 ± 1.6 ###
18:3 *n*-6	2.2 ± 1.5	1.7 ± 2.3	2.2 ± 1.3	1.0 ± 0.8 #	2.0 ± 1.5	2.4 ± 0.4	2.4 ± 0.4
18:3 *n*-3	1.7 ± 1.2	3.1 ± 1.5 #	1.0 ± 0.3 ***	4.2 ± 2.9 #	2.0 ± 1.3 *	1.5 ± 0.2	1.3 ± 0.4
20:0	1.3 ± 0.8	1.6 ± 1.4	0.9 ± 0.4	2.2 ± 2.2	1.7 ± 2.3	0.6 ± 0.1 #	0.5 ± 0.2 #
20:1 *n*-9	1.7 ± 1.9	1.5 ± 1.9	2.1 ± 2.2	1.2 ± 0.5	1.6 ± 0.8	0.4 ± 0.1 #	0.4 ± 0.2 #
20:2 *n*-6	0.9 ± 1.1	0.5 ± 0.2	0.8 ± 0.5	0.6 ± 0.5	0.5 ± 0.3	0.2 ± 0.1 #	0.2 ± 0.1
20:4 *n*-6	1.4 ± 0.7	2.2 ± 1.6	1.4 ± 0.5	2.4 ± 1.2 #	2.9 ± 2.3	1.6 ± 0.2	1.3 ± 0.3 **
20:5 *n*-3	2.1 ± 1.5	1.9 ± 1.7	1.3 ± 0.7	2.6 ± 1.3	1.4 ± 0.8 *	2.8 ± 0.3	2.5 ± 0.6
Others	1.4 ± 1.4	3.5 ± 2.3 #	2.8 ± 0.5 ##	2.4 ± 1.5	3.3 ± 2.3 #	0.7 ± 0.2	0.8 ± 0.2
SFA	50.9 ± 9.6	40.8 ± 7.6 #	42.7 ± 4.9 #	43.4 ± 7.7	47.8 ± 7.4	37.3 ± 2.2 ###	40.3 ± 2.6 *##
MUFA	34.1 ± 6.5	37.5 ± 5.0	36.8 ± 4.5	37.7 ± 8.4	32.5 ± 4.8	32.8 ± 1.7	30.7 ± 1.9 *
PUFA *n*-6	9.8 ± 3.5	15.9 ± 8.4 #	14.8 ± 4.1 #	9.3 ± 2.5	12.7 ± 3.8 *	25.0 ± 1.4 ###	24.4 ± 1.4 ###
PUFA *n*-3	3.7 ± 2.4	4.3 ± 3.3	3.9 ± 1.7	7.3 ± 3.6	4.5 ± 2.9	4.2 ± 0.3	3.8 ± 0.8

^a^ Data are presented as mg/100 mg of triglycerides. Abbreviations: MB, Mediterranean breakfast; WB, Western breakfast.; SFA, saturated fatty acid; MUFA, monounsaturated fatty acid; PUFA, polyunsaturated fatty acid. Values are expressed as mean ± standard deviation. N = 12. * *p* < 0.05, ** *p* < 0.01, and *** *p* < 0.001 vs. MB; # *p* < 0.05, ## *p* < 0.01, ### *p* < 0.001 vs. control. *p*-values are Bonferroni-corrected for the multiple comparisons.

## Data Availability

Data is provided within the manuscript or Supplementary Information Files.

## Supplementary Materials

The following supporting information can be downloaded at: https://www.mdpi.com/article/10.3390/nu18040672/s1, Supplementary Table S1 with the nutritional composition of both experimental meals and Supplementary Table S2 with the sequences of the primers.

## Abbreviations

ApoB	Apolipoprotein B
BMI	Body mass index
CD36	Cluster of Differentiation 36
CM	Chylomicrons
CONSORT	Consolidated Standards of Reporting Trials
CVD	Cardiovascular Disease
DBP	Diastolic blood pressure
DNA	Deoxyribonucleic Acid
ELISA	Enzyme-Linked Immunosorbent Assay
ER	Endoplasmic Reticulum
FA	Fatty acid
FAME	Fatty acid methyl esters
FID	Flame ionization detector
GC	Gas chromatography
GAPDH	Glyceraldehyde-3-phosphate dehydrogenase
HDL-c	High-density lipoprotein cholesterol
HOMA-IR	Homeostatic Model Assessment of Insulin Resistance
LAM	Lipid-Associated Macrophage
LC-Diol	Liquid Chromatography-Diol
LDL-c	Low-density lipoprotein cholesterol
LDLR	Low-density lipoprotein receptor
LPL	Lipoprotein Lipase
LRP1	Low-density lipoprotein receptor-related protein 1
MB	Mediterranean-style breakfast
MCP-1	Monocyte Chemoattractant Protein 1
mRNA	messenger RNA
MUFA	Monounsaturated fatty acid
PMA	Phorbol 12-myristate 13-acetate
PL	Phospholipid
PUFA	Polyunsaturated fatty acid
RT-qPCR	Reverse Transcription quantitative Polymerase Chain Reaction
SBP	Systolic blood pressure
SFA	Saturated fatty acid
SPE	Solid-phase extraction
SRB1	Scavenger receptor class B type 1
SRA2	Scavenger receptor class A type 2
SD	Standard Deviation
TG	Triglyceride
TRL	Triglyceride-rich lipoprotein
WB	Western-style breakfast
WHO	World Health Organization
VLDL	Very low-density lipoprotein
