# Supplementary material for: A Western-Style Breakfast Induces a More Pro-Inflammatory Postprandial Response and Promotes Greater Macrophage Lipid Accumulation Compared to a Mediterranean-Style Breakfast in Obese and Normal-Weight Individuals
Source: Nutrients. 2026 Feb 18;18(4):672. doi: 10.3390/nu18040672 (PMC12943205; doi:10.3390/nu18040672)
Supplement: Supplementary file 1 [file nutrients-18-00672-s001.zip › nutrients-4068901-supplementary.pdf]

**Supplementary Table 1. Nutritional analysis of Mediterranean and Western-style breakfasts.**

| Parameter | Mediterranean-style breakfast | Western-style breakfast |
|---|---|---|
| **Energy (kJ)** | 2022 | 1963 |
| **Energy (kcal)** | 485 | 471 |
| **Protein (g)** | 15 | 14 |
| **Carbohydrates (g)** | 62 | 53 |
| **Sugars (g)** | 36 | 26 |
| **Total fat (g)** | 57 | 53 |
| **- Saturated fat (g)** | 8 | 32 |
| **- Monounsaturated fat (g)** | 44 | 15 |
| **- Polyunsaturated fat (g)** | 5 | 2 |
| **Cholesterol (mg)** | 0 | 104 |
| **Fiber (g)** | 7 | 6.4 |

**Supplementary Table 2. Primer Sequences and Amplicon Characteristics for Gene Expression Analysis of Lipoprotein Receptors by qPCRa**

| Gene | GenBank™ Accession | Forward and Reverse Primers (5'–3') | Nucleotides Location | Amplicon Size (bp) |
|---|---|---|---|---|
| **LDLr** | AY114155 | F: TGAAGTTGGCTGCGTTAATGTG | 812–833 | 69 |
| | | R: CATTCGCCGCTGTGACACT | 862–880 | |
| **LRP** | NM_002332 | F: CCGGAGTGGTATTCTGGTATAAGC | 13779–13802 | 126 |
| | | R: CTCCGCCTTCGTACATCTTGTA | 13883–13904 | |
| **VLDLr** | D16493 | F: ATGTTATCCAGCGTGGACTTGA | 1937–1958 | 74 |
| | | R: GGATGAGCTAGGAACTCCAGAGACT | 1986–2010 | |
| **SR-A2** | NM_002445 | F: GCCAACCTCATGGACACAGA | 519–538 | 79 |
| | | R: GCTGCAGAAGAATGTCATTAAATCTT | 572–597 | |
| **SR-B1** | BC080647 | F: TTCTACACTCAGCTGGTGTTGATG | 1415–1438 | 65 |
| | | R: AGCGCCAGGAGGAGCTACT | 1461–1479 | |
| **CD36** | L06850 | F: CTCTTTCCTGCAGCCCAATG | 468–487 | 75 |
| | | R: ACTGTGAAGTTGTCAGCCTCTGTT | 519–542 | |
| **GAPDH** | AF261085 | F: CAACGGATTTGGTCGTATTGG | 127–147 | 72 |

[a]Abbreviations: LDLr, low-density lipoprotein receptor; LRP, LDL receptor-related protein; VLDLr, very-low-density lipoprotein receptor; SR-A2, scavenger receptor class A type 2; SR-B1, scavenger receptor class B type 1; CD36, scavenger receptor CD36; GAPDH, glyceraldehyde-3-phosphate dehydrogenase.
